# Efficacy and safety of triple therapy versus dual antiplatelet therapy in patients with atrial fibrillation undergoing coronary stenting: A meta-analysis

**DOI:** 10.1371/journal.pone.0199232

**Published:** 2018-06-19

**Authors:** Liyao Liu, Jietao Huang, Xiaogang Zhang, Xiaoman Tang

**Affiliations:** 1 Department of Cardiology, The First Affiliated Hospital of Chongqing Medical University, Chongqing, P. R. China; 2 Department of Cardiology, Chongqing Emergency Medical Center, Chongqing, P. R. China; University of Tampere, FINLAND

## Abstract

**Background:**

The optimal antithrombotic therapy for atrial fibrillation (AF) patients undergoing coronary stenting is unknown. The present meta-analysis sought to investigate the efficacy and safety of triple therapy (TT; warfarin, clopidogrel and aspirin) vs dual antiplatelet therapy (DAPT; clopidogrel plus aspirin) in those patients.

**Methods:**

PubMed and Cochrane Library were searched for studies enrolling AF patients undergoing coronary stenting on TT and DAPT up to September 2016, and fourteen studies were included. Efficacy outcomes included ischemic stroke, stent thrombosis, major adverse cardiovascular event (MACE), all-cause mortality and myocardial infarction (MI); safety outcome was major bleeding. We conducted meta-analysis and used odds ratio (OR) with 95% confidence intervals (CI) to compare TT and DAPT. Meta-regression, sensitivity and subgroup analysis were taken to investigate the source of heterogeneity in the outcome of major bleeding.

**Results:**

14 eligible observational studies with 11,697 subjects were identified. Compared with DAPT, TT had decreased the risk of ischemic stroke [OR = 0.74, 95% CI (0.59, 0.93), P = 0.009] and stent thrombosis [OR = 0.40, 95% CI (0.18, 0.93), P = 0.033]. While, there was an increased risk of major bleeding [OR = 1.55, 95% CI (1.16, 2.09), P = 0.004] associated with TT. The risk of MACE, all-cause mortality and MI had no significant statistical difference between TT and DAPT. Furthermore, the results of univariate and multivariate meta-regression analysis implicated that there were no obvious correlations between certain baseline characteristics (age, gender, race, hypertension, study design) and risk of major bleeding. Also of major bleeding, the findings of sensitivity analysis were generally robust, and a prespecified subgroup analysis of race demonstrated that the source of heterogeneity might attribute to Asian studies mostly.

**Conclusions:**

TT reduced the risk of ischemic stroke and stent thrombosis with an acceptable major bleeding risk compared with DAPT, and TT was considered as a valid alternative in AF patients undergoing coronary stenting. Further prospective randomized trials are needed to ensure the reliability of these data and find the optimal therapeutic strategy in this setting of patients.

## Introduction

The current European Society of Cardiology (ESC) Guidelines [1] for the management of atrial fibrillation (AF) recommended the use of triple therapy (TT; warfarin, clopidogrel and aspirin) in the patients with AF undergoing coronary stenting (class IIa, level of evidence B or C). Oral anticoagulant (OAC) with warfarin significantly decreases the incidence of ischemic stroke and peripheral embolism in patients with high-risk AF [1]. Whereas the combination of aspirin and clopidogrel (dual antiplatelet therapy; DAPT) is the standard regimen for the prevention of recurrent coronary events in patients undergoing coronary stenting [2]. It is estimated that almost 10% of patients receiving percutaneous coronary intervention (PCI) were found to have a history of AF [1,3]. In clinical practice, there are various antithrombotic regimens (such as TT, DAPT, warfarin combined with a single antiplatelet agent, etc.) used in this setting of patients. However, the efficacy and safety of different antithrombotic regimens are still controversial. Recently, the PIONEER AF-PCI trial (An Open label, Randomized, Controlled, Multicenter Study Exploring Two Treatment Strategies of Rivaroxaban and a Dose-Adjusted Oral Vitamin K Antagonist Treatment Strategy in Subjects With Atrial Fibrillation Who Undergo Percutaneous Coronary Intervention) [4] demonstrated that among patients with AF undergoing intracoronary stent placement, rivaroxaban plus DAPT significantly reduced the risk of major bleeding compared with the vitamin K antagonist (VKA) plus DAPT, and the efficacy outcomes were comparable between two regimes. However, according to current AF guidelines the non-vitamin K antagonist oral anticoagulants (NOACs) are lack of evidence among certain patients, such as valvular-AF and prosthetic heart valves, and there are no effective antagonists at present. Moreover, in developing countries the availability and cost of NOACs are more problematic than expected. For that, warfarin appears still important in AF patients with coronary stent. We performed this updated meta-analysis of pertinent studies to assess the efficacy and safety of TT in patients with AF after coronary stenting compared with DAPT.

## Methods

### Literature search and selection

Pertinent English articles were searched in PubMed, and Cochrane Library up to September 2016. The language of the papers was restricted to English. These searches were supplemented by manual review of the guidelines and references of included articles. The systematic search strategy was showed in Supporting information (S1 File). Two independent investigators (LLY and HJT) screened the citations through the title and abstract. Studies were included if: (1) patients with AF undergoing coronary stenting. (2) clinical studies comparing TT with DAPT. (3) outcomes including major bleeding and ischemic stroke. The exclusion criteria were: (1) studies without real control group. (2) studies with duplication. (3) ongoing/ unpublished study. (4) less than 30 days follow-up. (5) review and meta-analysis. (6) to avoid double counting.

### Quality assessment and data extraction

We used the Newcastle-Ottawa scale [5] to evaluate the methodological quality of observational trials. This scale includes three factors: cohorts selection, comparability of cohorts, and assessment of outcome. The score ranges from 0 to 9 stars allocated to each study. Observational studies achieving six or more stars were considered to be of high quality. All included studies were independently collected by two investigators (LLY and HJT) after full-text review. Any disagreement was resolved by consensus with all the authors. The data extraction was collected: name of study, country, design, duration of follow-up, baseline demographics, and clinical outcomes at follow-up.

### Study endpoints

The endpoints of this meta-analysis included: (1) primary endpoints are efficacy outcomes: ischemic stroke, stent thrombosis, major adverse cardiovascular event (MACE), all-cause mortality, and myocardial infarction (MI); (2) secondary endpoint is safety outcome: major bleeding. We accepted the endpoint definitions adopted by the original articles, regardless of the slight difference of definition among studies. Therefore, the risk of MACE and ischemic stroke could be replaced by major adverse cardiac and cerebral events (MACCE) and stroke [6–11], if no relevant data existed. Two coauthors independently recorded the occurrence of the events above according to the original studies.

### Statistical analysis

Dichotomous variables were reported as odds ratio (OR), 95% confidence interval (CI). The I^2^ statistic and P value were used to measure the heterogeneity. If the I^2^ statistic ≥50% and P <0.05, we considered statistically significant heterogeneity among studies and used a random-effects model of meta-analysis; if not, a fixed-effect model was adopted in the statistical analysis. We performed Egger’s test to evaluate the publication bias, and P <0.1 regarded as significant asymmetry. Sensitivity analysis omitting one study at a time was conducted to assess the stability of the results. Meta-regression and subgroup analyses were carried out to investigate potential sources of heterogeneity which was quantitatively assessed by the I^2^. Age (<75 years and ≥75 years), male proportion, race (Caucasian and Asian), hypertension patient proportion, and study design (retrospective and prospective) were included in the univariate analysis, while age, male and race were also involved in multivariate analysis. Race was included as a prespecified subgroup. Statistical significance was expressed as a two-tailed P value <0.05. Analyses were conducted by the means of STATA software version 12.0 (StataCorp, College Station, TX) in present article.

## Results

**Fig 1:**
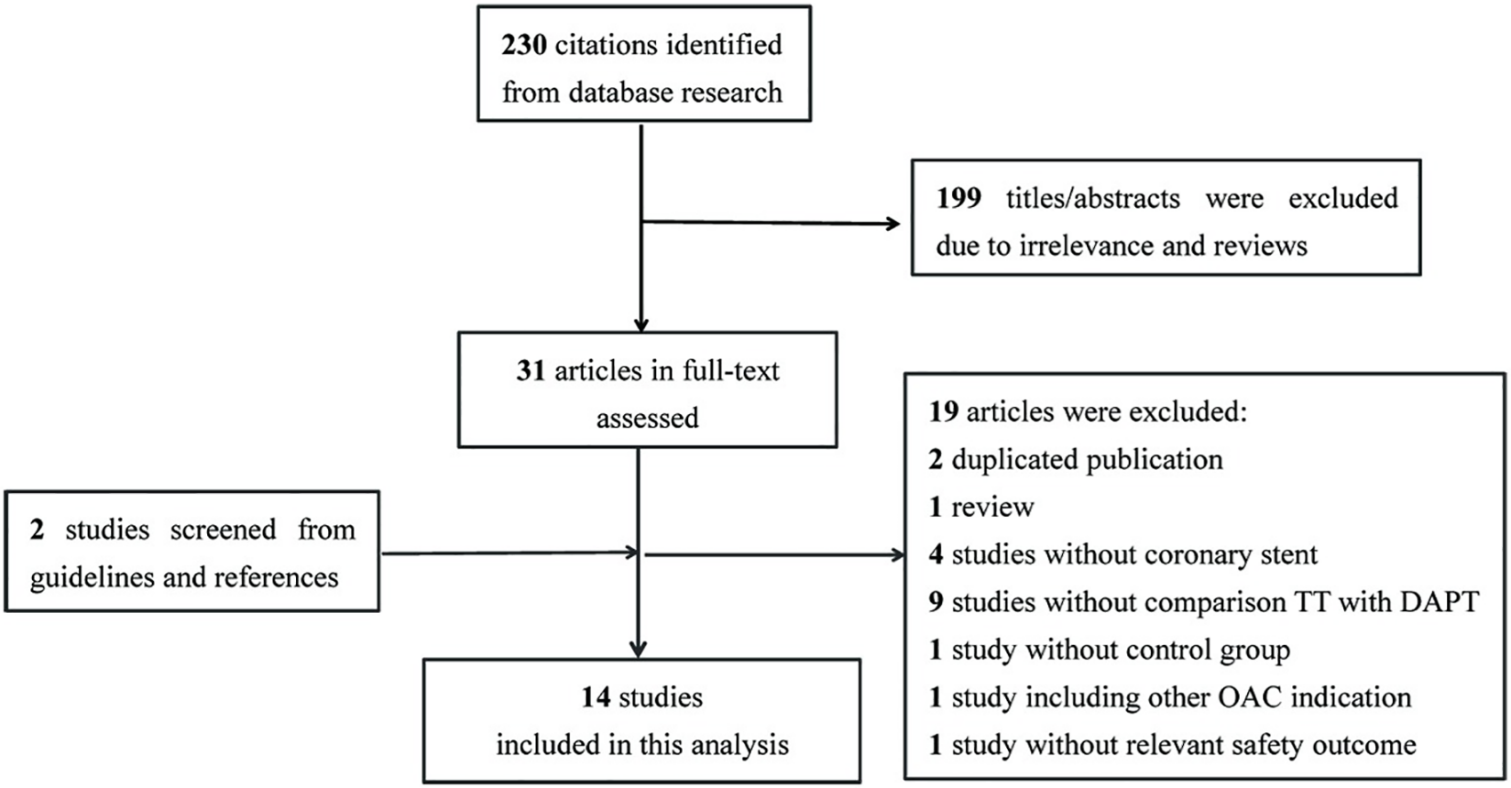
Flow diagram showing the process of study selection.

A total of 230 studies were collected after the initial search. Of them, 199 studies were excluded due to irrelevance and reviews by screening of titles and abstracts, another 19 studies were excluded according to the criteria by full-text review, and 2 additional studies were identified through other resources. 14 eligible studies [6–19] including 9 retrospective observational studies and 5 prospective observational studies finally remained (Fig 1).

These fourteen studies enrolled 11,697 subjects with AF undergoing coronary stenting discharged with different antithrombotic therapies: 4,266 patients received TT and 7,431 received DAPT. The follow-up of included studies ranged from 0.5 year to 5 years, the median was 1 year. The scores of Newcastle-Ottawa scale of included studies ranged from six to eight stars, which were considered as high quality. The detailed description is displayed in Table 1.

**Table 1:** General features of included studies.

Study	Country	Design	Follow-up	Patients and regimen	INR and TTR in TT	major bleeding	NOS
Dąbrowska et al., 2013	Poland	Prospective*	12 months	18 TT vs 29 DAPT	2.0–2.5; NS	Nonstandard definition	******
De Vecchis et al., 2015	Italy	Retrospective	378 ± 15.7 days	48 TT vs 19 DAPT	NS; NS	Nonstandard definition	*******
Fosbol et al., 2013	Denmark	Retrospective*	12 months	448 TT vs 1200 DAPT	NS; NS	ICD-9 codes	******
Gao et al., 2010	China	Prospective	12 months	136 TT vs 334 DAPT	1.8–2.5; NS	TIMI	*******
Goto et al., 2014	Japan	Prospective*	Median 5.1 years	286 TT vs 551 DAPT	1.6–2.6; 52.6%	GUSTO TIMI	*******
Hess et al., 2015	USA	Retrospective*	24 months	1370 TT vs 3589 DAPT	NS; NS	ICD-9 codes	********
Ho et al., 2012	Canada	Retrospective	5.9 ± 5.0 months	382 TT vs 220 DAPT	2.0–2.5; NS	Nonstandard definition	******
Kang et al., 2015	Korea	Retrospective*	24 months	131 TT vs 236 DAPT	2.0–3.0; 29.20 ± 24.88%	GUSTO	*******
Kawai et al., 2014	Japan	Retrospective	Median 37 months	28 TT vs 67 DAPT	NS; NS	TIMI	******
Maegdefessel et al., 2008	Germany	Retrospective*	Median 1.4 years	14 TT vs 103 DAPT	NS; NS	Nonstandard definition	******
Mennuni et al., 2015	USA, Italy	Retrospective*	12 months	371 TT vs 488 DAPT	NS; NS	BARC	*******
Rubboli et al., 2014	Europe	Prospective*	12 months	679 TT vs 162 DAPT	2.0–3.0; NS	BARC	********
Sambola et al., 2016	Spain	Prospective*	12 months	318 TT vs 267 DAPT	2.0–2.5; NS	TIMI BARC	*******
Suh et al., 2013	Korea	Retrospective	42.0 ± 29.0 months	37 TT vs 166 DAPT	1.83 ± 0.41; NS	Intracranial bleeding	*******

TT, triple antithrombotic therapy; DAPT, dual antiplatelet therapy; INR, international normalized ratio; TTR, times in therapeutic range; ICD, International Classification of Diseases; TIMI, Thrombolysis in Myocardial Infarction; BARC, Bleeding was defined according to the Bleeding Academic Research Consortium; GUSTO, Global Utilization of Streptokinase and Tissue Plasminogen Activator for Occluded Coronary Arteries classification; NS, not stated; NOS, Newcastle-Ottawa scale, observational studies achieving 6 or more * were considered to be of high quality;

* Registry.

Baseline characteristics of patients are showed in Table 2. Median patient age was 72 years (68 to 78), 70% (45% to 75%) being man. There were 81% (70% to 94%) hypertension, 35% (27% to 41%) diabetes mellitus, 67% (23% to 96%) dyslipidemia, 13% (10% to 19%) previous stroke, 26% (15% to 56%) heart failure, 29% (4% to 49%) previous MI, 29% (4% to 62%) chronic renal failure. We conducted subanalyse in the outcome of major bleeding regard to the race: 9 studies for Caucasian and 5 studies for Asian.

**Table 2:** Baseline characteristics of patients.

Study	Male	Mean age (years)	Hypertention	Diabetes Mellitus	Dyslipidemia	Previous stroke	Heart failure	Previos MI	Chronic renal failure
Dąbrowska et al., 2013	59%	70	88%	40%	96%	10%	NS	41%	NS
De Vecchis et al., 2015	45%	73	78%	35%	55%	13%	15%	25%	29%
Fosbol et al., 2013	58%	78	81%	34%	57%	13%	22%	34%	NS
Gao et al., 2010	71%	71	70%	37%	68%	14%	21%	18%	27%
Goto et al., 2014	71%	73	85%	34%	NS	19%	40%	12%	NS
Hess et al., 2015	58%	78	82%	32%	64%	11%	19%	29%	NS
Ho et al., 2012	71%	72	79%	35%	76%	12%	49%	NS	4%
Kang et al., 2015	65%	68	75%	31%	44%	14%	26%	8%	10%
Kawai et al., 2014	73%	72	87%	41%	68%	19%	NS	49%	56%
Maegdefessel et al., 2008	74%	69	90%	27%	68%	10%	NS	NS	NS
Mennuni et al., 2015	71%	73	94%	41%	NS	12%	54%	NS	62%
Rubboli et al., 2014	70%	73	84%	36%	67%	17%	20%	25%	NS
Sambola et al., 2016	75%	73	75%	38%	55%	15%	56%	33%	16%
Suh et al., 2013	63%	68	70%	36%	23%	14%	27%	4%	10%

MI, myocardial infarction; NS, not stated.

### Efficacy outcomes

#### Ischemic stroke

**Fig 2:**
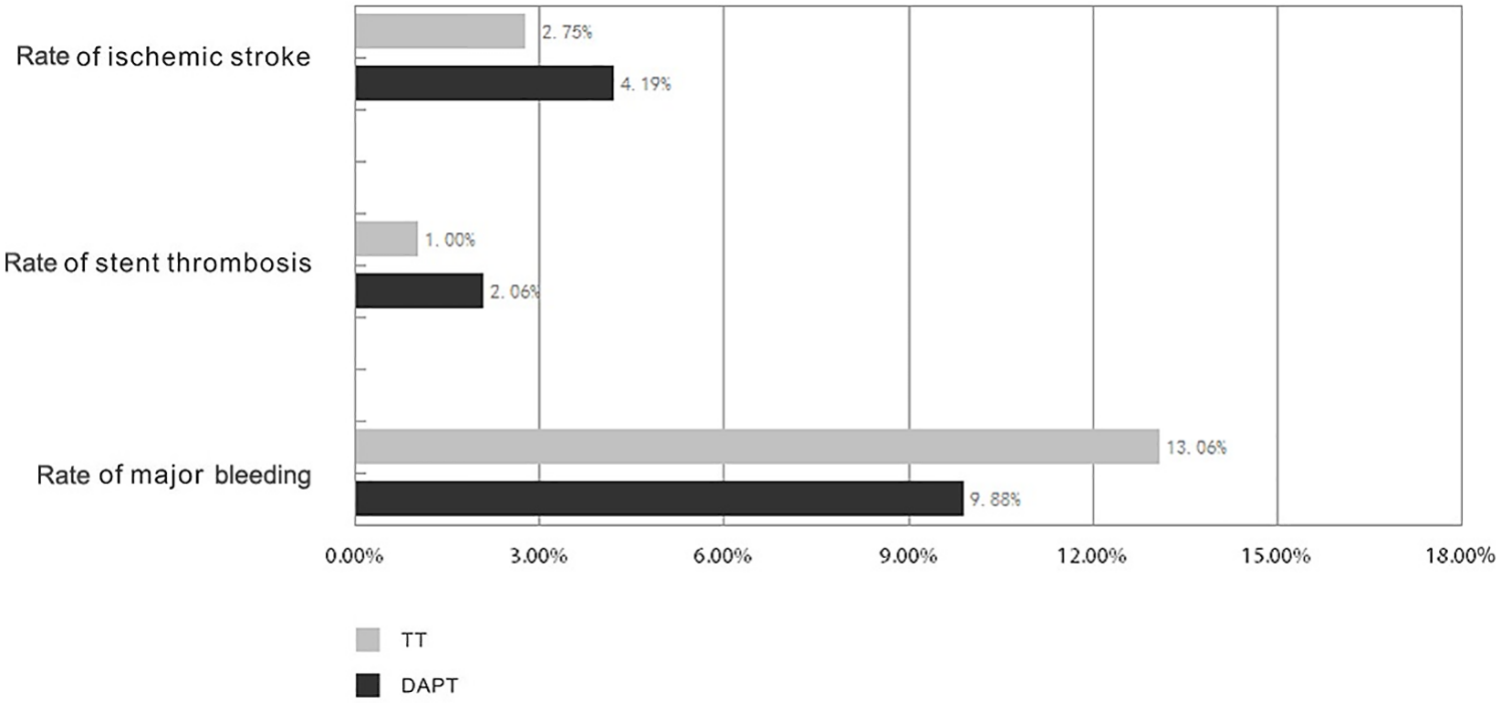
Rates of ischemic stroke, stent thrombosis and major bleeding at a follow up of median 1 year.

**Fig 3:**
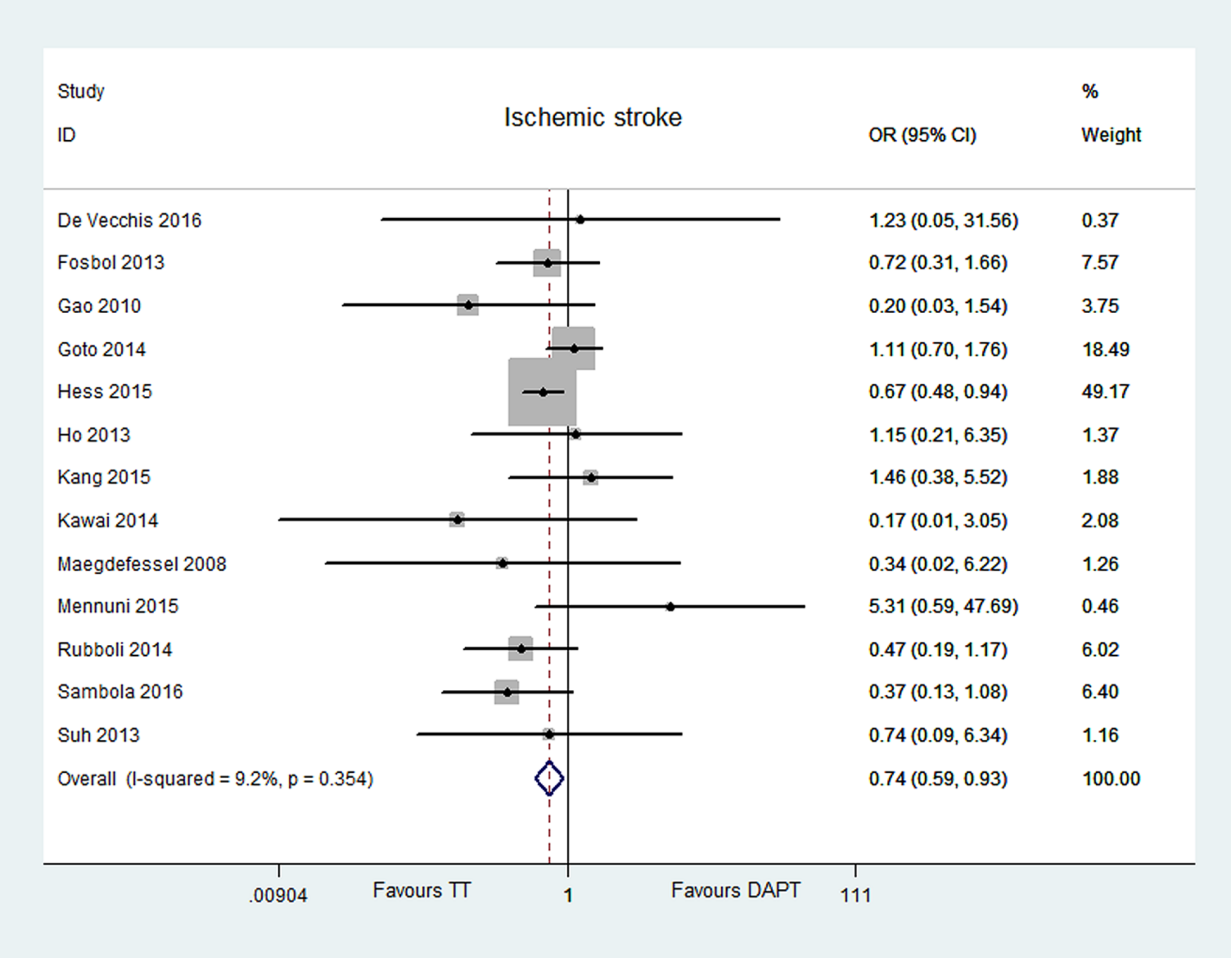
Forest plot of ischemic stroke in TT group vs DAPT group.

Thirteen articles reported the outcome of ischemic stroke [6–11, 13–19]. Ischemic stroke were reported in 2.75%, for 4248 patients receiving TT, and in 4.19%, for 7402 patients receiving DAPT. TT had a 26% reduced risk of ischemic stroke compared with DAPT [OR = 0.74, 95% CI (0.59, 0.93), P = 0.009]. The I^2^ was 9.2% and P = 0.354 indicating no significant heterogeneity in this analysis. No publication bias were found (Egger’s test P = 0.716). The data have shown in Figs 2 and 3.

#### Stent thrombosis

**Fig 4:**
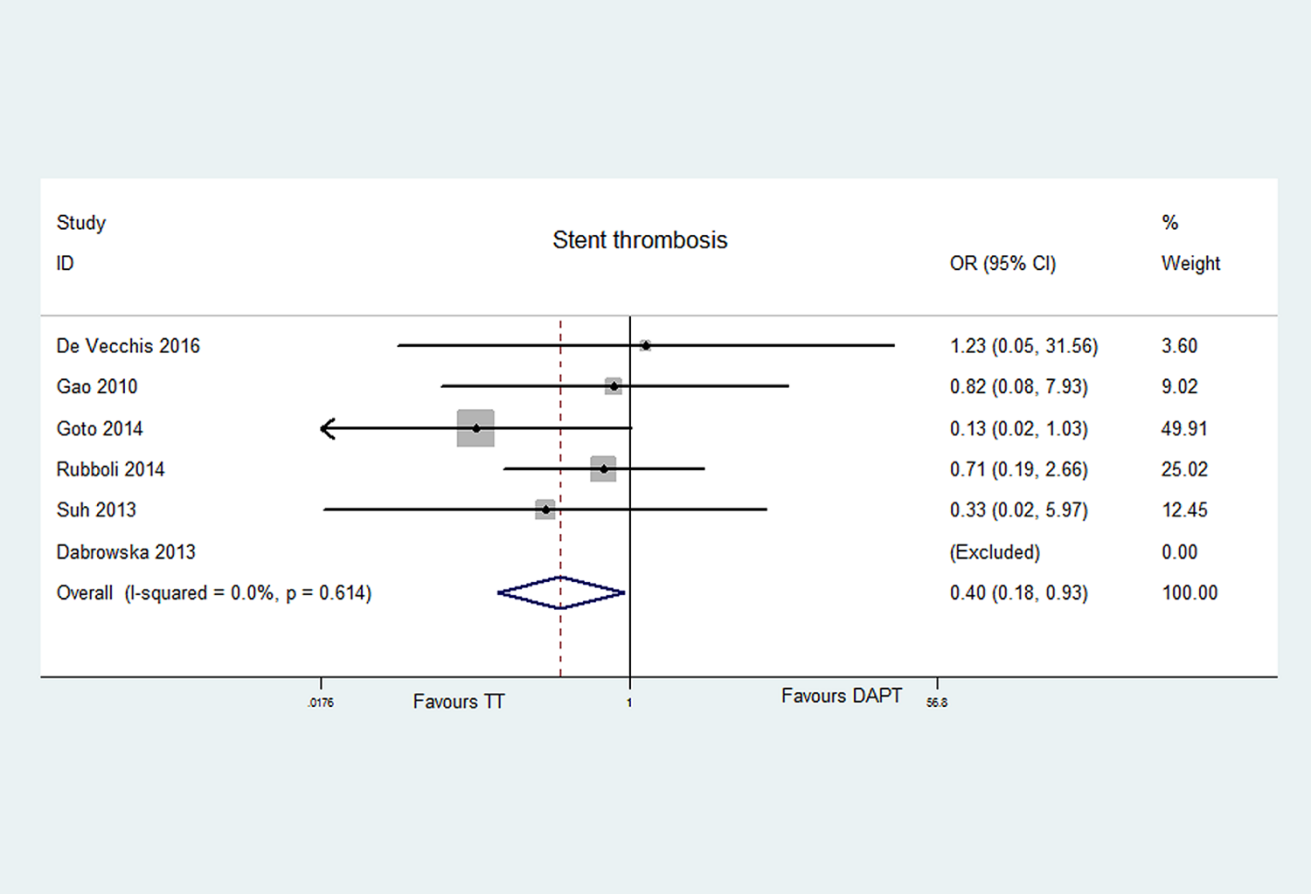
Forest plot of stent thrombosis in patients with TT and DAPT.

The data of stent thrombosis were reported in six articles [6, 9, 11–13, 15]. Totally, the rate of stent thrombosis in 1204 patients on TT was 1.00%, and that in 1261 patients on DAPT was 2.06%. TT had significantly decreased the risk of stent thrombosis with 60% [OR = 0.40, 95% CI (0.18, 0.93), P = 0.033], compared with DAPT. No statistical heterogeneity was found (P = 0.614, I^2^ = 0.0%). No publication bias was noted (Egger's P = 0.852). The data are displayed in Figs 2 and 4.

#### MACE, all-cause mortality, and MI

**Fig 5:**
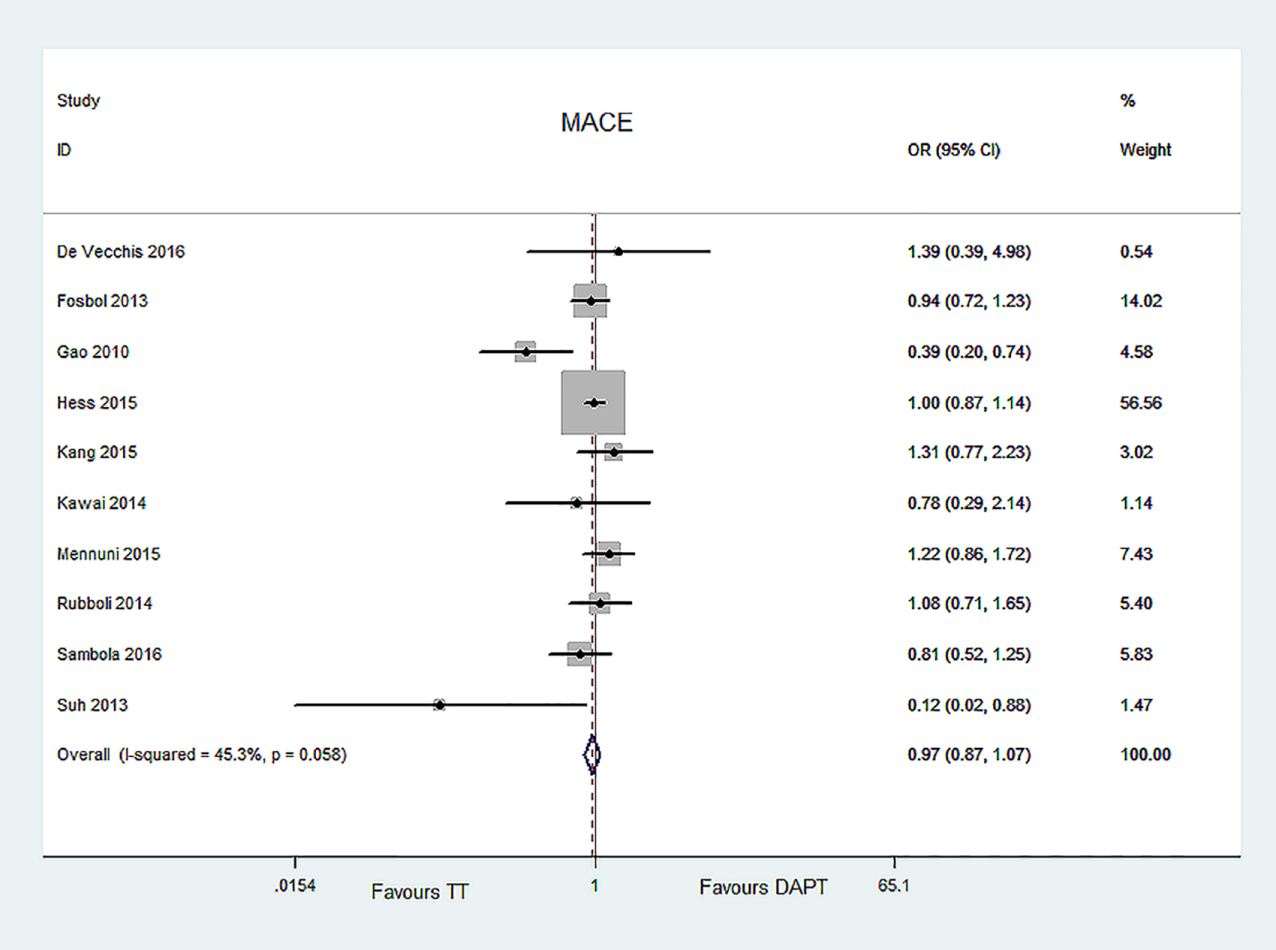
Forest plot of MACE in TT group and DAPT group.

**Fig 6:**
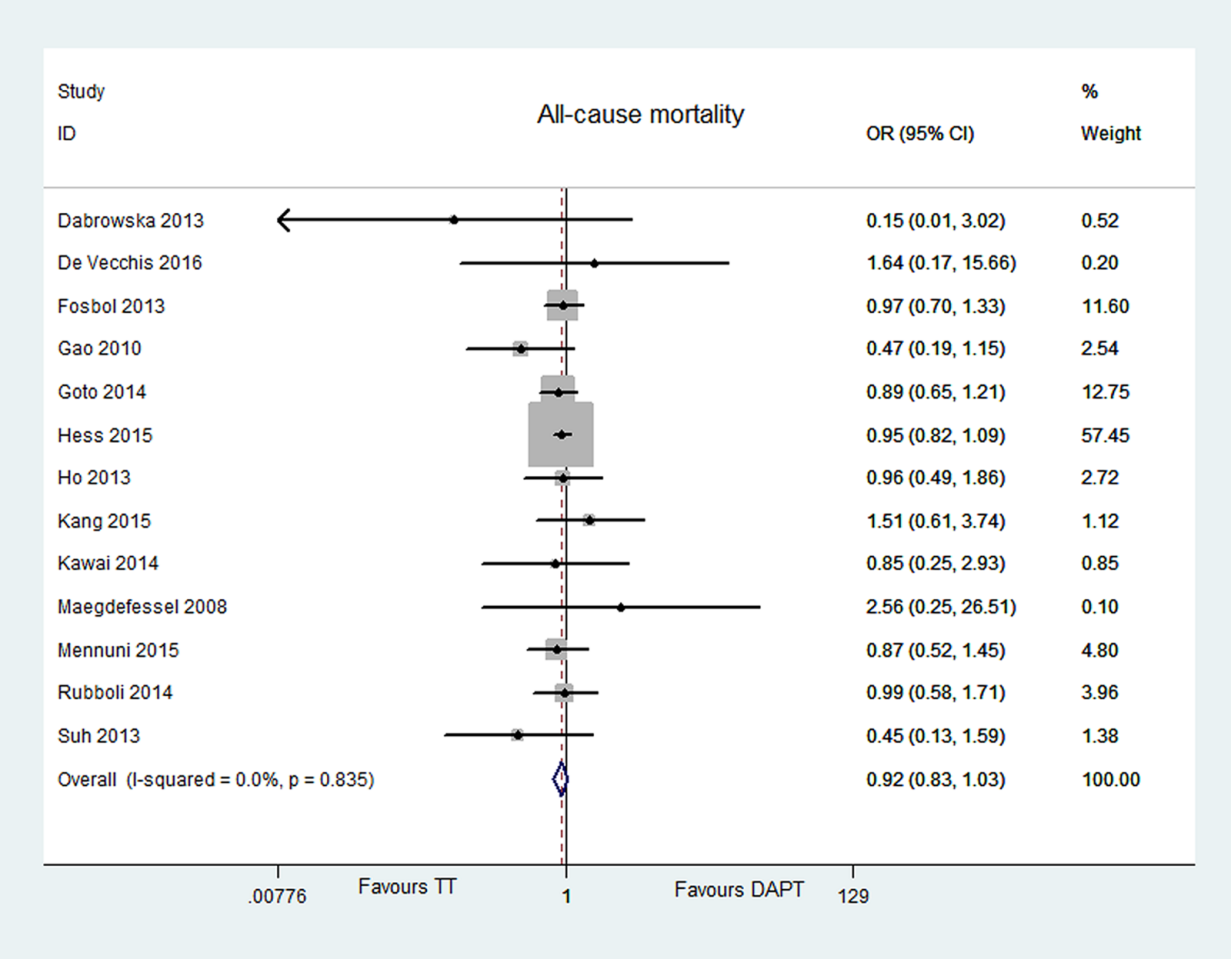
Forest plot of all-cause mortality in TT group vs DAPT group.

**Fig 7:**
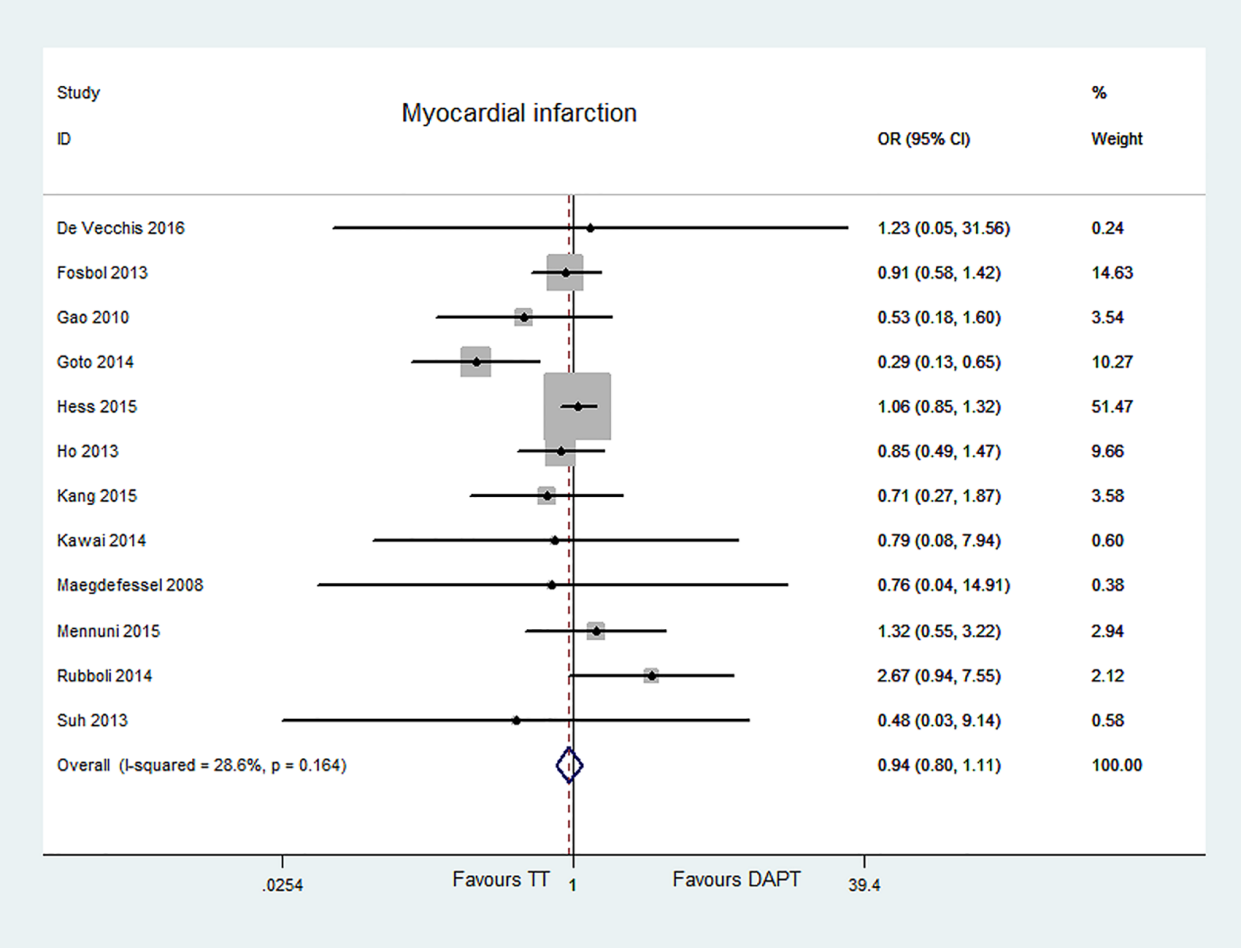
Forest plot of MI in patients with and DAPT.

Ten articles reported MACE [6–11, 13, 14, 16, 18], thirteen articles for all-cause mortality [6–9, 11–19], twelve studies for MI [6–9, 11, 13–19]. The risk of MACE, all-cause mortality, and MI did not significantly differ between two groups [MACE, OR = 0.97, 95% CI (0.87, 1.07), P = 0.508; all-cause mortality, OR = 0.92, 95% CI (0.83, 1.03), P = 0.165; MI, OR = 0.94, 95% CI (0.80, 1.11), P = 0.487]. No evident heterogeneity or publication bias was found [MACE, P = 0.058, I^2^ = 45.3%, Egger’s test P = 0.302; all-cause mortality, P = 0.835, I^2^ = 0.0%, Egger’s test P = 0.49; MI, P = 0.164, I^2^ = 28.6%, Egger’s test P = 0.424]. The data were showed in Figs 5–7.

### Safety outcomes

#### Major bleeding

**Fig 8:**
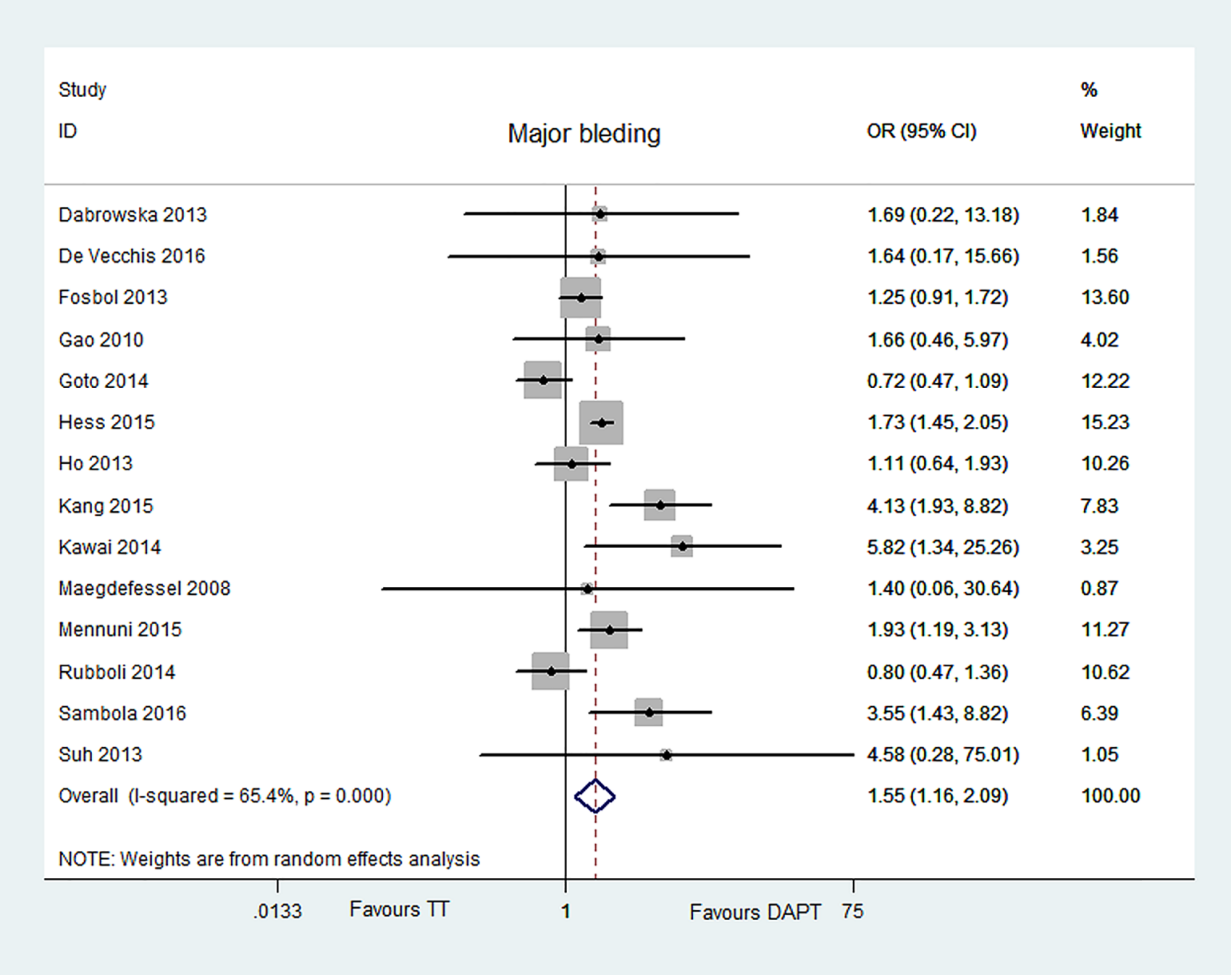
Forest plot of major bleeding in patients with TT vs DAPT.

Major bleeding was reported in 14 articles [6–19]. Overall, Major bleeding were reported in 13.06% for 4266 patients on TT, and in 9.88% for 7431 patients on DAPT. TT was associated with a 1.55-fold increased risk [OR = 1.55, 95% CI (1.16, 2.09), P = 0.004], compared with DAPT. However, the heterogeneity between the two groups was significant (P = 0.000, I^2^ = 65.4%), and we had performed meta-regression, sensitivity and prespecified subgroup analyses in order to find the source of heterogeneity. No publication bias for major bleeding was found with the Egger’s test P = 0.666. The data are illustrated in Figs 2 and 8.

### Meta-regression, sensitivity and subgroup analyses

**Fig 9:**
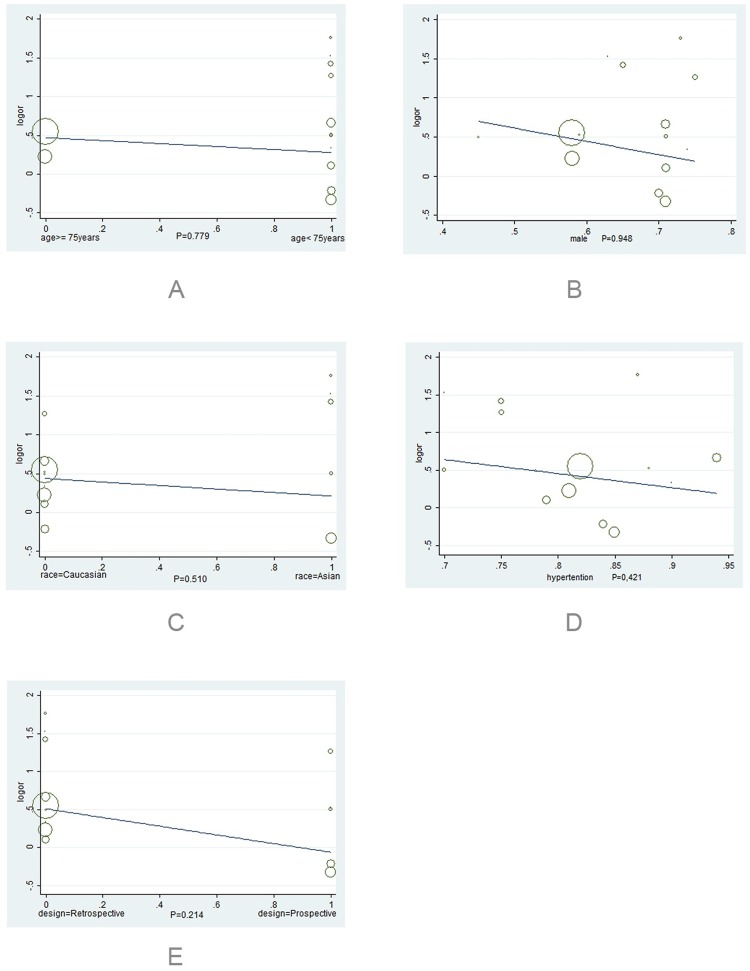
Scatter plots of univariate regression analysis in major bleeding. (A) The proportion of age (<75 years, ≥75 years); (B) Male proportion; (C) The proportion of race (Caucasian and Asian); (D) Hypertension patient proportion; (E) study design (retrospective and prospective).

**Fig 10:**
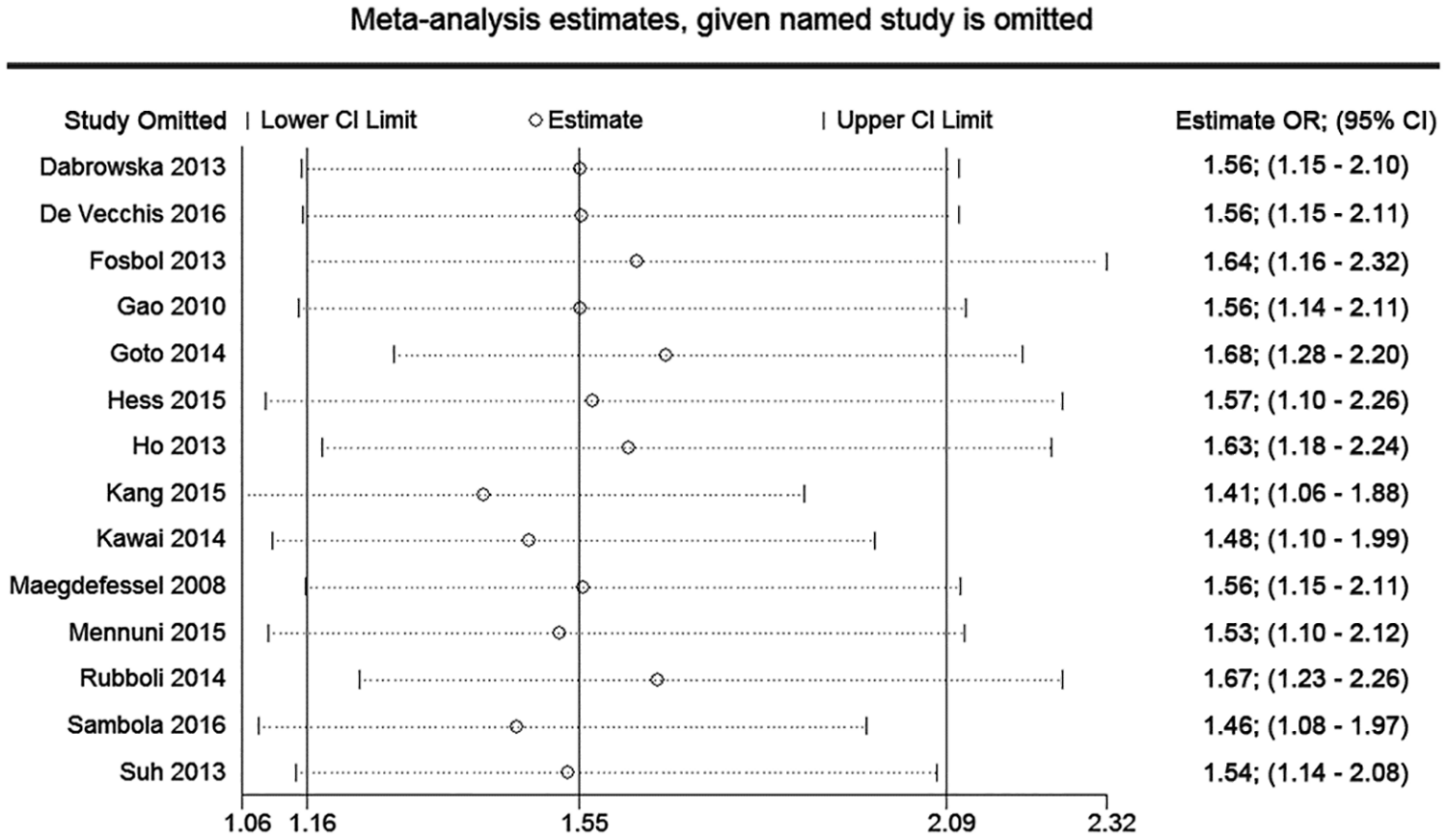
Plot of sensitivity analysis in major bleeding.

**Fig 11:**
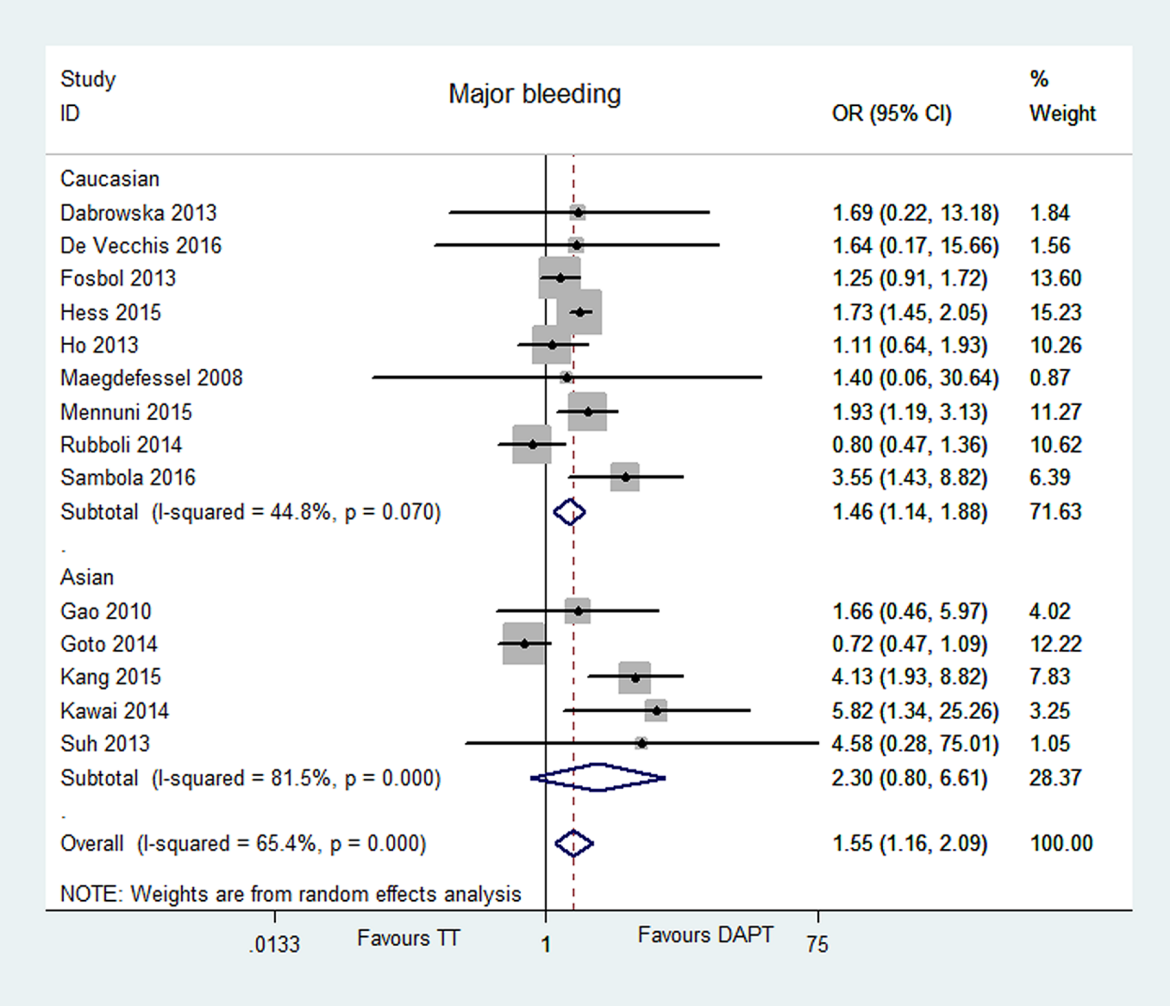
Forest plot of subgroup analysis with Caucasian and Asian in major bleeding.

Our univariate regression analyses showed that there were no significant correlations between age (P = 0.779), male proportion (P = 0.948), race (P = 0.510), hypertension patient proportion (P = 0.421), study design (P = 0.214) and the outcome of major bleeding. The results of multivariate regression analysis implicated that there were no statistically significant relationship between age (P = 0.900), male proportion (P = 0.887), race (P = 0.548) and the risk of major bleeding. We then performed a sensitivity analysis excluding one study in each turn, and the findings were generally robust. In the outcome of major bleeding, Asian subgroup involving 5 studies, there was no statistically significant difference between TT and DAPT [OR = 2.30, 95% CI (0.80, 6.61), P = 0.123], and significant statistical heterogeneity was found (P = 0.000, I^2^ = 81.5%). Whereas, Caucasian subgroup enrolled nine studies, and compared with DAPT, TT had a 1.46-fold increased risk of major bleeding [OR = 1.46, 95% CI (1.14, 1.88), P = 0.003] with no significant heterogeneity (P = 0.070, I^2^ = 44.8%). The results of meta-regression are showed in Table 3. The data are showed in Figs 9–11.

**Table 3:** Meta-regression of major bleeding.

Risk factor	Univariate	Multivariate
	P value	P value
Age (<75 years, ≥75 years)	0.779	0.900
Male proportion	0.948	0.887
Race (Caucasian, Asian)	0.510	0.548
Design (retrospective, prospective)	0.214	NA
Hypertension patient proportion	0.421	NA

NA, not available.

## Discussion

Our study sought to investigate efficacy and safety of additional OAC in the AF patients post coronary stenting. The main findings of this present analysis are as follows: compared with DAPT, TT reduced the risk of ischemic stroke and stent thrombosis with increased major bleeding risk in this setting of patients, and the risk of MACE, all-cause mortality and MI was comparable.

At present, the optimal antithrombotic therapy for AF patients receiving coronary stenting is unknown. Several studies [8,14,15,20] demonstrated that in real-world practice most AF patients undergoing coronary stent placement received DAPT rather than TT at discharge, which was contrary to the current recommendation [1] and led to occurrence of stroke. In other words, OAC was underused in those patients, for fear of bleeding complication in clinical practice. From existing evidence, a retrospective study from CRUSADE Registry (Can Rapid Risk Stratification of Unstable Angina Patients Suppress Adverse Outcomes with Early Implementation of the American College of Cardiology/American Heart Association Guidelines) [14] indicated that TT had a similar ischemic event risk versus DAPT but a trend toward increased bleeding among elderly non–ST-segment elevation myocardial infarction (NSTEMI) patients with AF undergoing coronary stenting. In addition, a meta-analysis by Chaofei Chen et al. [21] also confirmed the increased major bleeding risk and non decreased ischemic stroke risk as to TT. However, in that meta-analysis the subjects who required OAC treatment were not only AF patients, but also prosthetic heart valves, pulmonary embolism, etc. Different anticoagulation indications have inconsistent risk of thrombosis, which may result in various outcomes [22]. Therefore, the efficacy and safety of TT and DAPT regimen need to be further evaluated in patients post coronary stenting requiring anticoagulation therapy only with AF. For this reason, We performed the present analysis and we had different findings.

Oral anticoagulation therapy has shown clear superiority in stroke prophylaxis over DAPT in the Atrial Fibrillation Clopidogrel Trial With Irbesartan for Prevention of Vascular Events (ACTIVE—W) study [23]. Moreover, a prospective study of Gao fei et al. [6] indicated that warfarin was more effective for stroke prevention in Asian population than that in white population. The results from our study showed that compared with DAPT, TT had reduced 26% risk of ischemic stroke among AF patients who underwent coronary stenting. Therefore, a including Asian study [6] demonstrated apparent benefits of TT in stroke prevention, especially in AF patient after coronary stenting with CHADS_2_ (congestive heart failure, hypertension, age ≥ 75 years, diabetes mellitus, and stroke [doubled]) score ≥ 2, while a including Caucasian study [10] indicated that TT decrease the thromboembolism risk in this setting of patients with CHA_2_DS_2_-VASc (cardiac failure or dysfunction, hypertension, age ≥ 75 years [doubled], diabetes mellitus, and stroke [doubled]–vascular disease, age 65–74 years, and sex category [female]) score ≥ 2. The aim of TT use in AF patient with coronary stent is to prevent systematic thromboembolic events. Previous study [24] demonstrated that TT had reduced stent thrombosis rates to 1% in the first month and to 1–2% in the first year among AF patients with coronary stent. Our meta-analysis showed that TT was associated with a 60% decreased risk of stent thrombosis, compared with DAPT. Summarily, from our results, TT could significantly reduce the risk of ischemic stroke and stent thrombosis in AF subjects undergoing coronary stenting.

From our results, TT was associated with 1.55-fold increased risk of major bleeding, which was lower than 2-5-fold risk reported in several studies [25, 26]. Especially, TT did not increase the risk of MACE and all-cause mortality irrespective of the increased major bleeding risk. These were in contrast with previous study [27] which reported that major bleeding were the most important cause of mortality among patients who underwent coronary stenting requiring oral anticoagulation therapy. In terms of the incidence of major bleeding in our analysis, it was difficult to compare the absolute rate among studies owing to the different bleeding definitions, follow-up duration and comorbidities in each studies. Besides, a target international normalized ratio (INR) between 2.0–2.5 in this setting of patients on TT, which could significantly reduce the major bleeding, was recommended by several studies [11, 15, 17, 25, 28]. Moreover, a prospective multicenter registry [10] demonstrated that in non-valvular AF patient undergoing PCI with CHA2DS2-VASc scores = 1, TT was associated with a high risk of bleeding without a significant benefit in thromboembolism prevention. Another included study [17] also suggested benefits of TT with CHADS_2_>2. On the other hand, TT had a lower mortality risk in AF patient post coronary stenting even with a HAS-BLED score of 3 or higher [12, 25], and the choice of OAC may accord to the risk of thromboembolism [8].

In order to explore the source of heterogeneity in the outcome of major bleeding, we conducted meta-regression including univariate and multivariate analysis, which showed those factors (age, male proportion, race, hypertension patient proportion and study design) were not the source of heterogeneity. The sensitivity analysis by omitting one study in each turn confirmed the robustness of our results. We then performed a prespecified subgroup analysis according to the findings of RE-LY (Randomized Evaluation of Long-Term Anticoagulant Therapy) trial [29], and we found that the source of heterogeneity was attributable to Asian studies mainly [OR = 2.30, 95% CI (0.80, 6.61), P = 0.123]. All the included fourteen studies were observational studies in present analysis, and only two studies [7, 15] reported times in therapeutic range (TTR), which were both from Asian subgroup, and whose TTR were at relatively low level (showed in Table 1). Furthermore, the RE-LY trial [29] revealed that the control of INR and mean TTR varied in different regions: TTR approached 62.4% in Western Europe, 50.9% in North America, but only between 32% and 40% in China and Southeast Asia. Moreover, TTR was proved in AF patients with coronary stent as a strong indicator of probability for both bleeding and thromboembolism risk, which should be maintained at high level [15, 30–32]. Therefore, we suppose that in this present analysis, a relative lower quality of TTR in Asian group might be a confounding factor, which led significant heterogeneity in total outcome of major bleeding. Besides, there might be other factors identified by the AF guideline [1] resulting in heterogeneity in present meta-analysis, including clinical presentation (category of AF, acute coronary syndrome, stable coronary artery disease, multivessel disease, etc.), prior stroke, renal dysfunction, stent type, Procedural characteristics of coronary intervention, CHA2DS2-VASc scores, target INR, individual TTR, duration of TT.

Recently, D’Ascenzo et al. [33] reported a similar meta-analysis which had different findings. The study involved patients with an indication for oral anticoagulants who underwent coronary stenting, and tested TT vs DAPT (9 studies), TT vs OAC and clopidogrel (6 studies). The primary endpoint was major bleeding, secondary ones were all-cause death, MI, stent thrombosis, and stroke, and the author assessed the secondary ones in summery OR. Then, this study concluded that compared to TT, both DAPT and OAC and clopidogrel were associated with reduced bleeding risk, and no increased risk of major adverse cardiac events (death, MI, stroke, and stent thrombosis). However, there were no detail results for ischemic events (stroke and stent thrombosis), which were meaningful and important in clinical practice. Hence, in that study we questioned the efficacy of both DAPT and OAC and clopidogrel in reducing the risk of stroke compared with TT.

Unfortunately, we did not evaluate the effect of combination with warfarin and clopidogrel (dual therapy, DT) due to scarce data deriving from included observational studies. The What Is the Optimal Antiplatelet and Anticoagulant Therapy in Patients With Oral Anticoagulation and Coronary Stenting (WOEST) study [34] revealed that OAC plus clopidogrel reduced bleeding events without increasing the risk of stroke and stent thrombosis. However, in that study the indications of anticoagulation were not only AF, but also pulmonary embolism, heart aneurysm, and that could be a potential confounding factor for assessing the effect of DT use in AF patients. Recently, the PIONEER AF-PCI trial including 2124 patients demonstrated that compared with TT, rivaroxaban combined with DAPT had lower bleeding events in AF patients who underwent coronary stenting, but the study was underpowered for ischaemic endpoints [4, 35]. Besides, there are three ongoing large-scale studies, which evaluate combinations of Edoxaban, Dabigatran and Apixaban with antiplatelet therapy in AF patients undergoing coronary stenting (NCT02866175, NCT02164864 and NCT02415400) [35]. NOACs have the potential to replace warfarin in triple therapy use according to current review [25], but we still lack of substantial evidence at present. Further randomized trials are needed to assess the efficacy and safety of various regimens, such as DT, combination of NOAC and antiplatelet therapy in AF patients undergoing coronary stenting, and define the detailed impact of relative factors to maximize the benefits for patients.

There are several potential limitations in our analysis. First, all the including studies were observational studies, thus, we could not adjust our analysis and weigh role of stent type, duration of TT, CHA_2_DS_2_-VASc score, individual TTR of patients, which could be potential confounding factors and affect clinical outcomes. Second, due to the scarce data, we ignored the clinical presentation and indication of coronary stent, the use of TT in stable coronary artery disease and acute coronary syndromes may result in different effects. Third, the definition of major bleeding varies across the studies, which may increase the risk of bleeding. Moreover, we used MACCE and stroke replace MACE and ischemic stroke in few articles because of no relevant data existed, which could increase the rate of endpoint.

## Conclusions

In conclusion, our study revealed that compared with DAPT, TT reduced the risk of ischemic stroke and stent thrombosis with an acceptable risk of increasing major bleeding in AF patients undergoing coronary stenting. TT was considered as a valid alternative in those patients, which was in consistent with current guideline in this issue. However, it is crucial that further prospective randomized trials are needed to ensure the reliability of these data and find the optimal therapeutic strategy in this setting of patients.

## Supporting information

S1 Table PRISMA 2009 checklist. (PDF)

S1 File Supplemental systematic search strategy in PubMed. (PDF)
