# Supplementary material for: Efficacy and safety of triple therapy versus dual antiplatelet therapy in patients with atrial fibrillation undergoing coronary stenting: A meta-analysis
Source: PLoS One. 2018 Jun 19;13(6):e0199232. doi: 10.1371/journal.pone.0199232 (PMC6007837; doi:10.1371/journal.pone.0199232)
Supplement: S1 File — (PDF) [file pone.0199232.s002.pdf]

Supplementary File 1: Systematic search strategy in PubMed.

(((((((((((((((((((Inhibitors, Indirect Thrombin[Title/Abstract]) OR Thrombin Inhibitors, Indirect[Title/Abstract]) OR Anticoagulant Agents[Title/Abstract]) OR Agents, Anticoagulant[Title/Abstract]) OR Anticoagulant Drugs[Title/Abstract]) OR Drugs, Anticoagulant[Title/Abstract]) OR Indirect Thrombin Inhibitors[Title/Abstract])) OR "Anticoagulants"[Mesh])) OR (((((((((((((((((((Blood Platelet Antagonists[Title/Abstract]) OR Antagonists, Blood Platelet[Title/Abstract]) OR Aggregation Inhibitors, Platelet[Title/Abstract]) OR Inhibitors, Platelet Aggregation[Title/Abstract]) OR Blood Platelet Antiaggregants[Title/Abstract]) OR Antiaggregants, Blood Platelet[Title/Abstract]) OR Platelet Antiaggregants[Title/Abstract]) OR Antiaggregants, Platelet[Title/Abstract]) OR Blood Platelet Aggregation Inhibitors[Title/Abstract]) OR Platelet Inhibitors[Title/Abstract]) OR Inhibitors, Platelet[Title/Abstract]) OR Antiplatelet Agents[Title/Abstract]) OR Agents, Antiplatelet[Title/Abstract]) OR Antiplatelet Drugs[Title/Abstract]) OR Drugs, Antiplatelet[Title/Abstract]) OR Platelet Antagonists[Title/Abstract]) OR Antagonists, Platelet[Title/Abstract])) OR "Platelet Aggregation Inhibitors"[Mesh])) OR ((((((Thrombolytic Agents[Title/Abstract]) OR Antithrombic Drugs[Title/Abstract]) OR Antithrombotic Agents[Title/Abstract]) OR Fibrinolytic Drugs[Title/Abstract]) OR Thrombolytic Drugs[Title/Abstract])) OR "Fibrinolytic Agents"[Mesh])) OR (("Aspirin"[Mesh]) OR ((((((((((((((((((Zorprin[Title/Abstract]) OR Acetysal[Title/Abstract]) OR Acetylsalicylic Acid[Title/Abstract]) OR Acid, Acetylsalicylic[Title/Abstract]) OR 2-

AND (Acetyloxy) AND benzoic Acid[Title/Abstract]) OR Acylpyrin[Title/Abstract]) OR Aloxiprimum[Title/Abstract]) OR Colfarit[Title/Abstract]) OR Dispril[Title/Abstract]) OR Easprin[Title/Abstract]) OR Ecotrin[Title/Abstract]) OR Endosprin[Title/Abstract]) OR Magnecyl[Title/Abstract]) OR Micristin[Title/Abstract]) OR Polopirin[Title/Abstract]) OR Polopiryna[Title/Abstract]) OR Solprin[Title/Abstract]) OR Solupsan[Title/Abstract]))) OR ((((((((((((((((((((((Estedi Brand of Warfarin Sodium[Title/Abstract]) OR 4-Hydroxy-3- AND (3-oxo-1-phenylbutyl) AND -2H-1-benzopyran-2-one[Title/Abstract]) OR Coumadine[Title/Abstract]) OR Bailly Brand of Warfarin Sodium[Title/Abstract]) OR Apo-Warfarin[Title/Abstract]) OR Apotex Brand of Warfarin Sodium[Title/Abstract]) OR Gen-Warfarin[Title/Abstract]) OR Genpharm Brand of Warfarin Sodium[Title/Abstract]) OR Warfant[Title/Abstract]) OR Antigen Brand of Warfarin Sodium[Title/Abstract]) OR Coumadin[Title/Abstract]) OR Goldshield Brand of Warfarin Sodium[Title/Abstract]) OR Marevan[Title/Abstract]) OR Boots Brand of Warfarin Sodium[Title/Abstract]) OR Bristol-Myers Squibb Brand of Warfarin Sodium[Title/Abstract]) OR Warfarin Potassium[Title/Abstract]) OR Potassium, Warfarin[Title/Abstract]) OR Warfarin Sodium[Title/Abstract]) OR Sodium, Warfarin[Title/Abstract]) OR Aldocumar[Title/Abstract]) OR Aldo Brand of Warfarin Sodium[Title/Abstract]) OR Tedicumar[Title/Abstract])) OR "Warfarin"[Mesh])) OR ((vitamin k antagonist[Title/Abstract]) OR antagonist, vitamin k[Title/Abstract])) OR ((((((((((((((((PCR 4099[Title/Abstract]) OR PCR-4099[Title/Abstract]) OR

clopidogrel napadisilate[Title/Abstract]) OR clopidogrel, AND (S) AND -isomer[Title/Abstract]) OR Iscover[Title/Abstract]) OR BMS brand 2 of clopidogrel bisulfate[Title/Abstract]) OR clopidogrel hydrochloride[Title/Abstract]) OR clopidogrel-Mepha[Title/Abstract]) OR Plavix[Title/Abstract]) OR BMS brand 1 of clopidogrel bisulfate[Title/Abstract]) OR SC 25989C[Title/Abstract]) OR SC 25990C[Title/Abstract]) OR SR 25989[Title/Abstract]) OR clopidogrel besylate[Title/Abstract]) OR clopidogrel Sandoz[Title/Abstract]) OR clopidogrel bisulfate[Title/Abstract])) OR "clopidogrel"[Supplementary Concept]))) AND (((clinical[Title/Abstract] AND trial[Title/Abstract]) OR clinical trials as topic[MeSH Terms] OR clinical trial[Publication Type] OR random*[Title/Abstract] OR random allocation[MeSH Terms] OR therapeutic use[MeSH Subheading]))) AND (((((((((Fibrillation, Auricular[Title/Abstract]) OR Fibrillations, Auricular[Title/Abstract]) OR Atrial Fibrillations[Title/Abstract]) OR Fibrillation, Atrial[Title/Abstract]) OR Fibrillations, Atrial[Title/Abstract]) OR Familial Atrial Fibrillation[Title/Abstract]) OR Auricular Fibrillation[Title/Abstract]) OR Auricular Fibrillations[Title/Abstract])) OR "Atrial Fibrillation"[Mesh])) AND ((((((((coronary stent[Title/Abstract]) OR stent, coronary[Title/Abstract]) OR coronary stenting[Title/Abstract]) OR stenting, coronary[Title/Abstract]) OR coronary intervention[Title/Abstract]) OR intervention, coronary[Title/Abstract])) OR ((((((((((("Percutaneous Coronary Intervention"[Mesh]) OR Coronary Intervention, Percutaneous[Title/Abstract]) OR Coronary Interventions, Percutaneous[Title/Abstract]) OR Intervention, Percutaneous

Coronary[Title/Abstract]) OR Interventions, Percutaneous Coronary[Title/Abstract]) OR Percutaneous Coronary Interventions[Title/Abstract]) OR Percutaneous Coronary Revascularization[Title/Abstract]) OR Coronary Revascularization, Percutaneous[Title/Abstract]) OR Coronary Revascularizations, Percutaneous[Title/Abstract]) OR Percutaneous Coronary Revascularizations[Title/Abstract]) OR Revascularization, Percutaneous Coronary[Title/Abstract]) OR Revascularizations, Percutaneous Coronary[Title/Abstract]))
